# Contact Toxicity, Grain-Protective Efficacy, and Behavioral Effects of Methyl Benzoate and Acetophenone Against Four Major Stored-Product Insect Pests

**DOI:** 10.3390/insects17090958

**Published:** 2026-09-15

**Authors:** Gomaa R. M. Ramadan, Thomas W. Phillips, Mostafa A. I. Taha

**Affiliations:** 1 Department of Pesticide Chemistry and Technology, Faculty of Agriculture, Alexandria University, El-Shatby, Alexandria 21545, Egypt; mostafatahaa93@gmail.com; 2 Department of Entomology, Kansas State University, Manhattan, KS 66506, USA

**Keywords:** *Rhyzopertha dominica*, *Sitophilus oryzae*, *Tribolium castaneum*, *Trogoderma granarium*, contact toxicity, repellency, wheat grains

## Abstract

Although the fumigant activity of the naturally occurring compounds methyl benzoate and its analogues has been extensively evaluated against stored-product insect pests, their contact toxicity when applied as residual surface sprays and grain protectants, as well as their repellent effects, remains poorly studied. The current study evaluated the contact toxicity of methyl benzoate (MB) and a related natural product, acetophenone (AP), on glass surfaces and on wheat grains, along with their repellent activity, against adults of the lesser grain borer, *Rhyzopertha dominica*, the red flour beetle, *Tribolium castaneum*, the rice weevil, *Sitophilus oryzae*, and the khapra beetle, *Trogoderma granarium*. Both compounds were significantly toxic as residual films on glass surfaces, with *S. oryzae* and *T. granarium* being more susceptible to both compounds than *R. dominica* and *T. castaneum*. Dose-mortality studies found that the LC_50_ values for MB were 0.265, 0.233, 0.257, and 0.149 mg/cm^2^, while those for AP were 0.328, 0.156, 0.233, and 0.126 mg/cm^2^ for *R. dominica*, *S. oryzae*, *T. castaneum*, and *T. granarium* adults, respectively. Furthermore, both compounds were highly toxic to all insect species on wheat grain, achieving complete adult mortality at 2.0 g/kg after 3 days of exposure, with no adults emerging (0.0 adults) after 65 days of treatment and with no reduction in wheat grain weight (0.0% loss). However, MB and AP exhibited negligible to weak repellent or attractive effects toward all tested insect species, even at the highest concentration tested (50 µg/cm^2^). Overall, these findings suggest that MB and AP could be viable candidates for stored-product protection as residual applications and may serve as alternatives to, or reduce reliance on, synthetic insecticides.

## Simple Summary

The contact toxicity of the naturally occurring compounds methyl benzoate (MB) and its analogue acetophenone (AP) applied as residual surface sprays and grain protectants, and their repellent effects, remain insufficiently studied against stored-product insects. In the present study, we evaluated the contact toxicity of MB and AP applied as residual sprays on glass Petri dishes and as grain protectants applied directly to wheat kernels, as well as their repellent activity against *Rhyzopertha dominica*, *Tribolium castaneum*, *Sitophilus oryzae*, and *Trogoderma granarium.* Both compounds exerted significant toxicity against all insect of the species tested as residual films on glass Petri dishes, with *S. oryzae* and *T. granarium* being more susceptible to both compounds than *R. dominica* and *T. castaneum.* In addition, both compounds applied to wheat grains at 2.0 g/kg caused complete adult mortality of all insect species, with no adults emerging after 65 days of exposure and no loss in wheat grain weight. In contrast, MB and AP exhibited negligible to weak repellent or attractive effects toward all tested insect species. These findings suggest that MB and AP could be promising alternatives to, or reduce reliance on, synthetic insecticides to protect stored products from insect infestation.

## 1. Introduction

The food requirements for a rapidly increasing global human population have become a significant challenge for agriculture. The global population is expected to rise to approximately 9.1 billion people by 2050, requiring about 70% more food production to meet the future demand [1,2]. Worldwide, more than two billion tons of grains, including cereals, pulses, and oilseeds, are produced annually for direct human consumption and livestock feed to provide human nutritional needs [3,4]. To safeguard food security, cereal grains are stored at various stages in the supply chain from farm to consumer. During this annual progression from storage through processing, grains are exposed to severe post-harvest losses that range from 1 to 2% in developed countries to 20–50% in developing countries [5,6]. Infestations by stored-product pests, particularly insects, mites, bacteria, fungi, and rodents, are the main contributors to losses in stored grains and higher associated costs [7,8]. Among all, insects are considered the most important biotic factor responsible for 30–40% of losses in stored grains [9,10].

Disinfestation of stored grains relies heavily on fumigants such as phosphine and synthetic insecticides such as organophosphorus and pyrethroid compounds [11,12,13,14]. Although fumigants have a high impact, they are inefficient in non-airtight structures, offer only short-term protection, and reinfestation may occur after treatment if prevention is not assured. Moreover, the rise of phosphine resistance has become a global concern [14,15,16]. Additionally, the use of contact insecticides in stored grain protection has gradually declined because of increasing regulatory demands, growing consumer concerns about their residues, the evolution of insecticide resistance in a wide range of pests, and the high costs included in developing and registering new products [17,18,19]. Therefore, safer and more effective alternatives are urgently required to ensure the proper protection of stored grains.

Plant-based natural products have been extensively studied as low-risk alternatives to contact insecticides owing to their wide spectrum of biological effects. Among them, essential oils and their key compounds are very efficient against many pests and valued for their broad biological activity, low toxicity to mammals, environmental safety, and rapid degradation [20]. These natural products have been documented to exert significant activities, including contact toxicity, fumigant action, repellent activity, growth and development inhibition, oviposition deterrence, antifeedant activity, and ovicidal activity [21,22,23,24,25,26]. The volatile methyl ester methyl benzoate (MB) and its related ketone, acetophenone (AP), are naturally occurring volatiles found in a wide range of plant species, including snapdragon (*Antirrhinum* spp.), petunia (*Petunia* spp.), and alyssum (*Alyssum* spp.) [27,28,29,30,31,32]. MB occurs naturally in a wide range of plant tissues and is also emitted by plants in response to herbivore attack [33]. AP is primarily formed as a degradation product of MB and has been linked to the microbiota found in virus-infected mammals [34,35]. Both compounds have been approved for human safety as preservatives and components in fragrances, as well as additives in food, pharmaceuticals, and cosmetics, according to the US Food and Drug Administration (FDA) and European Union (EU) [36,37,38,39,40].

It has been previously documented that MB and AP showed significant fumigant toxicity against stored-product pests. For example, our previous report [41] concluded that MB and AP exerted fumigant activity against adults of *R. dominica* (Coleoptera: Bostrichidae), *T. castaneum* (Coleoptera: Tenebrionidae), and *S. oryzae* (Coleoptera: Curculionidae), and both compounds exhibited significant toxicity in the fumigation assay against cultures that contained mixed life stages of the tested species at 300 µL/L. Morrison et al. [38] noted that MB was highly efficient as a fumigant against *R. dominica* and *T. castaneum*. Among three benzoate compounds, MB was the most potent against phosphine-susceptible and phosphine-resistant strains of *S. oryzae* and *R. dominica* [42]. MB was also efficient in fumigation and repellency assays against the cigarette beetle, *Lasioderma serricorne* (Coleoptera: Anobiidae) [33]. Most previous studies have focused on fumigant activity, whereas only a few have investigated their contact toxicity as residual films on structural surfaces or as grain protectants against stored-product pests. For instance, Abdel-Baki et al. [43] assessed the contact toxicity of MB on dried beans against the dried-bean beetle, *Acanthoscelides obtectus* (Coleoptera: Chrysomelidae), and found that MB was very effective, inducing complete mortality at a rate of 5.4 g/kg with a 92.4% reduction in progeny production after 45 days of treatment.

Methyl benzoate and AP could function better as contact insecticides rather than as fumigants due to their low vapor pressure (0.38 mm Hg and 0.45 mm Hg for MB and AP, respectively, at 25 °C) [32]. Moreover, we observed in our earlier study [41] that there is difficulty in the volatility of MB and AP at the highest concentration (300 µL/L) used. Therefore, these considerations led us to conduct the current study to assess the contact toxicity of MB and AP as residual films applied to glass surfaces and as wheat grain protectants against the lesser grain borer, *R. dominica*, the rice weevil, *S. oryzae*, the red flour beetle, *T. castaneum*, and the khapra beetle, *T. granarium* (Coleoptera: Dermestidae). Additionally, the repellent activity of both compounds was investigated against the same four insect species.

## 2. Materials and Methods

### 2.1. Insects

The lab-reared strains of *R. dominica*, *T. castaneum*, *S. oryzae*, and *T. granarium* were maintained at the Bioassay Laboratory, Pesticide Chemistry and Technology Department, Faculty of Agriculture, Alexandria University, Alexandria, Egypt. Rearing conditions were maintained at 30 ± 2 °C with 50–70% R.H. and a 16:8 h light: dark cycle. *R. dominica*, *S. oryzae*, and *T. granarium* colonies were reared on uninfested winter wheat grains, while *T. castaneum* was fed a diet of 85% wheat flour, 10% oat flour, and 5% brewer’s yeast. Unsexed adults aged 1–4 weeks of *R. dominica*, *T. castaneum*, and *S. oryzae* and 1–3-day-old adults of *T. granarium* (a short-lived species) were used in the experiments.

### 2.2. Contact Toxicity of Methyl Benzoate and Acetophenone in Glass Petri Dishes Against Adult Beetles

Methyl benzoate and AP (purity for each at 99%) were obtained from Sigma-Aldrich, St. Louis, MO, USA. The residual film assay described in our previous study [44] was used to evaluate the toxicity of MB and AP against the selected beetles. Serial concentrations of each compound were prepared in acetone. The tested concentration range (0.08–0.5 mg/cm^2^) was selected based on preliminary experiments to provide an acceptable mortality range. A glass Petri dish (9 cm diameter; NORMAX, Lda., Marinha, Grande, Portugal) was used and considered an independent experimental replicate (experimental unit) for the contact toxicity assay. One mL of the given solution was evenly distributed onto the floor of each glass Petri dish to achieve the desired range of concentrations, and the solvent was allowed to evaporate entirely. To verify the complete evaporation of the acetone solvent at each concentration, a separate Petri dish treated with acetone only was used as a reference, and insects were introduced only after the solvent had fully evaporated. To stop adult insects from climbing the glass wall of the Petri dishes and escaping, polytetrafluoroethylene (quick-dry PTFE, from WaKooshi, Woking, Surrey, UK) was applied to the inner vertical rims of the dishes. Twenty mixed-sex adults of either *R. dominica*, *T. castaneum*, *S. oryzae*, or *T. granarium* were put into separate Petri dishes. All concentrations were tested in triplicate, with a total of 60 insects per concentration for each species. The control Petri dishes received acetone only. Petri dishes were partially covered, with the lids left slightly open at a consistent position across all replications to ensure ventilation and to prevent mortality related to the fumigant effects of MB and AP. Dishes were held at 25 ± 2 °C and 50–70% R.H and a photoperiod of 16:8 h (light:dark). Following 24 h of exposure, insect mortality was assessed under a microscope by gently moving individuals with a fine metal probe to confirm their response or movement. Insects that showed no response or movement were recorded as dead. Mortality in the control was less than 5.0%; therefore, no correction was applied before probit analysis. Following Finney [45], data for each species were then subjected to probit analysis to estimate the LC_50_ and LC_99_ values of MB and AP against the tested adult beetles.

### 2.3. Effectiveness of Methyl Benzoate and Acetophenone as Wheat Grain Protectants Against Adult Beetles

Treatments for *R. dominica*, *S. oryzae*, and *T. granarium* adults were conducted using 60 g of uninfested whole wheat grains. For *T. castaneum* adults, a diet of 90% whole wheat grains and 10% broken grains was employed. Sixty-gram portions were individually weighed and placed into a clean 1 L glass jar. The desired volumes of MB and AP were taken and mixed with 2 mL of acetone to prepare application rates of 0.25, 0.5, 1.0, and 2.0 g/kg, and the 2 mL of the corresponding solution was gradually pipetted onto the grains in each jar. Immediately after adding the MB and AP solution, jars were closed and hand-shaken for 2 min to ensure uniform coating of the grains. The lids were removed, and the jars were left open for 1 h to allow complete solvent evaporation. Control jars received acetone only. Following acetone evaporation, each 60 g portion was distributed into three 50 mL glass vials (20 g per vial). For each vial, 20 adults of each species were introduced separately. A muslin cloth, secured with a plastic ring, was used to cover the vials to ensure proper ventilation and to retain beetles in the vials. All vials were incubated under the same conditions used for rearing. Adult mortality of *R. dominica*, *T. castaneum*, and *S. oryzae* was evaluated at 3, 7, and 14 days, and at 3 and 7 days for *T. granarium*. Dead insects were removed at each interval, and surviving individuals were returned to the treated grains. Fourteen days post-treatment, dead and live adults of *R. dominica*, *T. castaneum*, and *S. oryzae* were separated from the grains using a sieve. The treated grains, including the infestation-related powders, were returned to the vials and held for an additional 51 days to allow for progeny development. For *T. granarium*, dead and live adults were sieved out after 7 days of treatment. Mortality percentages were computed for each treatment and corrected using the Abbott formula [46] when mortality in the corresponding control exceeded 5.0% for each insect species. Thereafter, adults emerging from treated grains following 65 days of exposure to varying concentrations of MB and AP were counted. The percentage reduction in adult emergence was computed using the following equation:

Reduction % = (A − B)/(A) × (100)

where A and B correspond to the total number of adults emerged in control and treatment, respectively.

To determine the grain weight loss, each vial was sieved to remove the fine powders resulting from insect infestation, and the remaining wheat grains in both the control and treatment vials were weighed. The weight loss percentage was calculated as:

Weight loss % = (Wi − Wf)/(Wi) × (100)

where Wi and Wf are the initial grain weight (20 g) and final grain weight, respectively, after the infestation-related powders have been sieved out.

### 2.4. Repellency Assay

The repellent activities of MB and AP were evaluated using the area-preference method as described in our previous study [44]. Different concentrations of MB and AP (6.25, 12.5, 25.0, and 50.0 µg/cm^2^) were prepared in acetone. For each replicate, one half of a Whatman No. 1 filter paper disc (9 cm diameter) was treated with 0.5 mL of the test solution, while the other half received only 0.5 mL of acetone. Treated halves were left to allow full evaporation of the solvent. Thereafter, both halves were secured together with adhesive tape and then placed at the base of a 9 cm glass Petri dish. Twenty adults of each species were individually released at the center of the Petri dish. The lids were used to cover the Petri dishes, leaving a slight gap to permit ventilation and prevent insect mortality due to the fumigant activity of MB and AP. To stop insect species from climbing the glass walls of Petri dishes and escaping, polytetrafluoroethylene was applied to the inner rims of the dishes. Concentrations were tested in triplicate. The Petri dishes were held at 30 °C, and adult counts were recorded on both halves at 0.5, 1.0, and 2.0 h after insect introduction. The repellency index (RI) was computed according to Mazzonetto and Vendramim [47], as RI = ($\frac{2Pt}{Pt+Pc}$), where Pt and Pc represent the percentage of insects recorded on the treated and control halves, respectively. The RI values below 1.0 classify the compound as repellent, values equal to 1.0 as neutral, and values above 1.0 as attractive.

### 2.5. Statistical Analysis

Probit analysis was employed to determine the LC_50_ and LC_99_ values for MB and AP. A one-way ANOVA was performed to analyze the data on progeny production and percentage weight loss in wheat grains within each insect species. Tukey HSD test was used for mean separations, and differences were considered significant at *p* < 0.05. For mortality data across exposure times in treated wheat grains, percentages were arcsine-transformed and analyzed using repeated-measures ANOVA, with application rate as the between-subjects factor and exposure time as the within-subjects (repeated) factor. For Repellency index (RI) data across exposure times, the same repeated-measures ANOVA was used without transformation. The main effects of application rate and exposure time, as well as their interaction, were evaluated for both mortality and RI. Sphericity was determined using Mauchly’s test, and the Greenhouse–Geisser adjustment was applied to correct for any violations. Pairwise comparisons among application rates within each insect species and at each exposure time were performed using Bonferroni adjustment, with differences considered significant at *p* < 0.05. Statistical analysis was performed using IBM SPSS V21.0 software (Statistical Package for Social Sciences, IBM Corp., Armonk, NY, USA).

## 3. Results

### 3.1. Contact Toxicity of Methyl Benzoate and Acetophenone Against Adult Beetles

Table 1 shows the probit parameters for adult mortality of selected insect adults exposed for 24 h at 25 °C to glass surfaces treated with different concentrations of MB and AP. Both compounds exhibited significant toxicity against adults of the four beetle species, with no significant differences observed between them, as indicated by the overlapping 95% confidence intervals of their LC_50_ values. *T. granarium* and *S. oryzae* adults exhibited greater susceptibility to both MB and AP compared to *R. dominica* and *T. castaneum*. The LC_50_ values for MB were 0.149 and 0.233 mg/cm^2^ for *T. granarium* and *S. oryzae*, compared with 0.126 and 0.156 mg/cm^2^ for AP, respectively. *R. dominica* and *T. castaneum* were more tolerant to both compounds, with LC_50_ values of 0.265 and 0.257 mg/cm^2^ for MB and 0.328 and 0.233 mg/cm^2^ for AP, respectively.

### 3.2. Mortality of Adult Beetles Exposed to Wheat Grains Treated with Methyl Benzoate and Acetophenone

The repeated-measures ANOVA revealed significant differences among application rates at each exposure time within each insect species. However, the main effect of exposure time and the application rate × exposure time interaction were nonsignificant in most cases (Table 2). MB exhibited significant efficacy against all tested adult beetles (Table 3). At 0.25 g/kg, MB exhibited limited toxicity against *R. dominica* and *S. oryzae* adults, with mortality rates of 23.3% and 27.5%, respectively, after 14 days of exposure. In contrast, MB caused high mortality in *T. castaneum* adults (91.7%) at the same rate. For *T. granarium*, MB produced significant efficacy, with 67.4% mortality after 7 days of exposure. At 0.5 g/kg, MB induced complete mortality (100.0%) in adults of *R. dominica* and *T. castaneum*. It also caused near-complete mortality in *S. oryzae* (86.3% after 14 days) and *T. granarium* (95.7% after 7 days). At application rates of 1.0 and 2.0 g/kg, MB achieved full mortality (100.0%) in all adult beetles.

Mortality percentages of selected adult beetles following exposure to wheat grains treated with AP at different application rates and exposure times are indicated in Table 4. The results showed that AP exerted low to moderate toxicity against adults of *R. dominica*, *S. oryzae*, and *T. castaneum* at 0.25 g/kg, with mortality rates of 0.0%, 5.9%, and 41.7%, respectively, after 14 days of exposure. For *T. granarium* adults, AP exhibited moderate to high toxicity, with mortality rates of 19.6% and 78.3% following 3 and 7 days of exposure, respectively. At 0.5 g/kg, mortality rates were 5.0%, 96.0%, and 78.3% for *R. dominica*, *S. oryzae*, and *T. castaneum*, respectively, after 14 days of exposure. For *T. granarium* adults, a mortality rate of 71.8% was recorded following a 7-day exposure period. At 1.0 g/kg, AP caused high mortality rates in all adult insect species, with values of 91.7%, 100.0%, and 98.3% for *R. dominica*, *S. oryzae*, and *T. castaneum*, respectively, after 14 days of exposure. For *T. granarium* adults, AP induced 93.5.0% mortality at 7 days post-treatment. At 2.0 g/kg, AP caused complete mortality (100.0%) in all adult beetles.

### 3.3. Effect of Methyl Benzoate and Acetophenone on Progeny Production of the Selected Beetle Species

Methyl benzoate and AP had significant effects in reducing the number of emerged adults of all tested insect species 65 days after treatment and adult infestation (Table 5). MB fully protected treated wheat grains, with no adults emerging (0.0 adults) from *T. castaneum* and *T. granarium* at all application rates. Complete inhibition of progeny production (0.0 adults) was also recorded for *R. dominica* and *S. oryzae* at application rates of 0.5, 1.0, and 2.0 g/kg, except for *S. oryzae* at 0.5 g/kg, whereby 4.7 adults emerged. Additionally, MB moderately reduced adult emergence of *R. dominica* and *S. oryzae* at 0.25 g/kg, with reduction percentages of 54.2% and 42.6%, respectively. A comparable effect was observed for AP, which significantly reduced adult emergence in all tested insect species. AP completely inhibited progeny production in *T. castaneum* and *T. granarium* at all application rates, except for *T. granarium* at 0.25 g/kg, which caused a 99.5% reduction (1.3 adults emerged). AP also exhibited high efficacy against *S. oryzae*, causing complete inhibition at 0.5, 1.0, and 2.0 g/kg, and an 82.8% reduction at 0.25 g/kg. For *R. dominica*, AP provided complete protection at 2.0 g/kg and significant reductions at 0.5 and 1.0 g/kg, with reduction percentages of 75.9% and 89.6%, corresponding to 42.3 and 18.3 adults emerging, respectively.

### 3.4. Effect of Methyl Benzoate and Acetophenone on Weight Loss and Damage in Treated Wheat Grains

**Figure 1:**
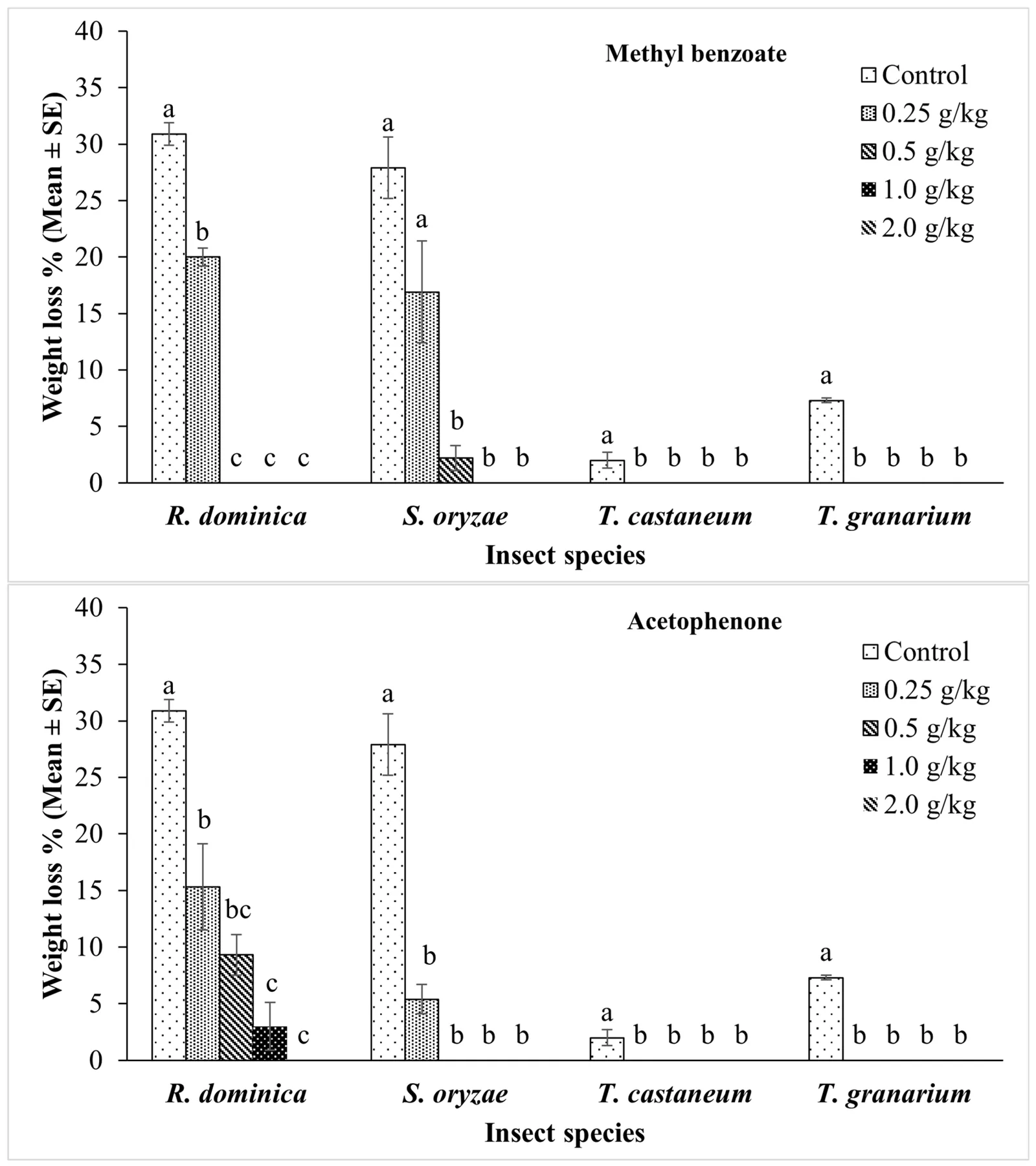
Weight loss percentages (mean ± SE) of wheat grains caused by insect infestation after 65 days of treatment with methyl benzoate and acetophenone at different application rates. Means followed by the same letter within each insect species show no significant difference (*p* > 0.05) according to ANOVA followed by Tukey HSD test.

Figure 1 shows the percentage weight loss in wheat grains resulting from insect infestation after 65 days of treatment with MB and AP at varying application rates. Both compounds significantly protected wheat grains against *T. castaneum* (F (4, 10) = 8.7, *p* = 0.003) and *T. granarium* (F (4, 10) = 1129.9, *p* < 0.001), with 0.0% weight loss recorded at all application rates, compared with 2.0% and 7.3% in the control treatments, respectively. For *S. oryzae*, both MB (F (4, 10) = 25.6, *p* < 0.001) and AP (F (4, 10) = 80.4, *p* < 0.001) significantly protected wheat grains, with complete protection (0.0% weight loss) at rates of 0.5, 1.0, and 2.0 g/kg, except for MB at 0.5 g/kg (2.2% weight loss), compared with 27.9% in the control. At the lowest rate (0.25 g/kg), treated wheat grains with MB and AP exhibited weight losses of 16.9% and 5.4%, respectively. For *R. dominica*, MB (F (4, 10) = 631.0, *p* < 0.001) provided complete protection at all application rates except at 0.25 g/kg (20.0% weight loss). In contrast, AP (F (4, 10) = 32.2, *p* < 0.001) achieved complete protection only at 2.0 g/kg. At 0.25, 0.5, and 1.0 g/kg, AP significantly reduced weight loss to 15.3, 9.3, and 3.0%, respectively, compared with 30.9% in the control.

### 3.5. Repellent Activity of Methyl Benzoate and Acetophenone Against Adult Beetles

The repeated-measures ANOVA revealed no significant differences among application rates at each exposure time within each insect species in most cases. Moreover, the main effect of exposure time and the application rate × exposure time interaction were nonsignificant in most cases (Table 6). Both MB and AP exerted negligible to low repellent effects against all beetle adults across all tested application rates (Table 7 and Table 8). MB was weakly attractive to *R. dominica*, *S. oryzae*, and *T. granarium*, except for *R. dominica* at 6.25 µg/cm^2^, while it exerted very weak repellent activity against *T. castaneum*. Overall RI values for MB ranged from 0.71 to 1.26 for *R. dominica*, 1.18 to 1.53 for *S. oryzae*, and 1.10 to 1.34 for *T. granarium*. Additionally, MB induced a very weak repellent effect against *T. castaneum*, with overall RI values ranging from 0.71 to 0.89. For AP, it showed weak repellency against *R. dominica* and *T. castaneum* adults at all application rates, with overall RI values ranging from 0.49 to 0.73. Additionally, AP exerted weak repellency against *T. granarium* adults at 25.0 and 50.0 µg/cm^2^, with overall RI values of 0.88 and 0.53, respectively. In contrast, AP exhibited weak attractiveness toward adults of *S. oryzae* at all tested application rates, with overall RI values greater than 1.0 (1.29 to 1.49), and toward *T. granarium* adults at 6.25 and 12.5 µg/cm^2^, with overall RI values of 1.18 and 1.01, respectively.

## 4. Discussion

Methyl benzoate and AP produced notable contact toxicity against all beetle adults tested in the present study, with *T. granarium* and *S. oryzae* adults being more susceptible to both compounds than *R. dominica* and *T. castaneum*. Limited studies have addressed the residual toxicity of both compounds against stored-product pests. However, our findings align with earlier studies reporting residual toxicity against stored-product insects. For example, Park et al. [48] showed that MB exhibited marked contact toxicity in a topical application assay against the azuki bean weevil, *Callosobruchus chinensis* (L.) (Coleoptera: Chrysomelidae), with an LC_50_ value of 44.81 µg/adult after 24 h of exposure. Acetophenone produced significant contact toxicity as a topical toxicant against *T. castaneum* (LD_50_ = 55.8 µg/adult) and *L. serricorne* (LD_50_ = 7.07 µg/adult) [49]. Additionally, Wang et al. [50] concluded that LC_50_ values of MB against larvae, pupae, and adults of *T castaneum* were 1.48, 0.49, and 0.77 mg/insect, respectively. For other pests, direct spraying of MB showed remarkable toxicity against the sweet potato whitefly, *Bemisia tabaci* (Gennadius) (Hemiptera: Aleyrodidae), yielding LC_50_ values of 0.3% for eggs and 0.2% for both fourth-instar nymphs and adults [51]. Mostafiz et al. [52] observed that MB produced the highest contact toxicity against nymphs and adults of the cotton aphid, *Aphis gossypii* Glover (Hemiptera: Aphididae), among three benzoate derivatives. MB at 1.0% caused 100.0% mortality in third-instar nymphs and adults of *A. gossypii*, with LC_50_ values of 0.18 and 0.32% after 24 h of application. Moreover, MB exhibited acaricidal toxicity against the two-spotted spider mite, *Tetranychus urticae* (Koch) (Acari: Tetranychidae), with LC_50_ values of 0.27 and 0.38% for eggs and adults, respectively [53]. Larson et al. [54] found that MB and AP displayed significant toxicity in a topical assay against females of the Egyptian mosquito, *Aedes aegypti* (Diptera: Culicidae), showing LD_50_ values of 45.6 and 18.4 µg/mosquito, respectively.

MB and AP exhibited effective activity as wheat grain protectants, resulting in 100.0% mortality against all adult beetles at application rates > 1.0%, with a 100.0% reduction in progeny production and full protection of wheat grains (0.0% weight loss). The present study is, to our knowledge, the first to evaluate the toxicity of MB and AP as grain protectants, except for Abdel-Baki et al. [43], who evaluated the toxicity of MB against *A. obtectus* in beans. The results of Abdel-Baki et al. [43] showed that MB at about 2.7 and 5.4 g/kg killed 81.0 and 100.0% of *A. obtectus* adults, with reductions of 77.3% and 92.4% in F1 progeny production after 45 days. Supporting our present findings, their results also revealed that MB lost its persistence in treated beans within the first three days, with no mortality observed by day 5. The present results revealed that the toxicity of MB and AP was independent of exposure time, as mortality did not increase with longer exposure periods. Additionally, the efficacy of MB and AP as grain protectants was also comparable to that of plant-based insecticides, including essential oils and their main components. For example, essential oils of *Psidium guajava*, *Haplophyllum tuberculatum*, and *Pinus roxburghii* killed 100% of *T*. *castaneum*, *S. oryzae*, and *T*. *granarium* adults, with a 100.0% reduction in progeny production at 5.0 g/kg [55]. Saad and Abdelgaleil [56] showed that (−)-terpinen-4-ol, α-terpinene, α-pinene, (−)-menthone, p-cymene, and (−)-citronellal at a rate of 5.0 g/kg caused mortality of 100.0, 100.0, 3.53, 100.0, 90.8, and 100.0% in *S. oryzae* adults in stored wheat, with inhibition of 100.0, 81.0, 33.87, 100.0, 100.0, and 100.0% in emerged adults, respectively.

Our results reveal that MB and AP can completely suppress progeny production, even though complete adult mortality was not achieved in some cases. Such effects may arise from the ability of both compounds to act as oviposition deterrents and possibly exert ovicidal effects, as previously observed for MB and AP against pests; however, these mechanisms were not directly investigated in the present study and require further investigation. For example, Mostafiz et al. [51] showed that application of MB at 1.0 and 2.0% using the leaf-dipping technique against the eggs of *B. tabaci* elicited reductions of 75.6 and 94.2% in egg hatch, respectively. Treatment of *T. urticae* eggs with MB at 0.5 and 1.0% in a leaf-dipping assay reduced the egg hatch rate by 76.9 and 92.5%, respectively [53]. Feng and Zhang [57] demonstrated that MB produced an ovicidal effect in a direct spray assay against the brown marmorated stinkbug, *Halyomorpha halys* (Hemiptera: Pentatomidae) (LC_50_ = 0.02 mg/cm^2^), the tobacco hornworm, *Manduca sexta* (Lepidoptera: Sphingidae) (LC_50_ = 0.015 mg/cm^2^), and the diamondback moth, *Plutella xylostella* (Lepidoptera: Plutellidae) (LC_50_ = 0.001 mg/cm^2^). MB was also able to deter oviposition in the fall armyworm, *Spodoptera frugiperda* (Lepidoptera: Noctuidae), with a reduction rate of 69.4% [58]. Mohsen and Ali [27] found that AP induced insecticidal and ovicidal effects against the southern house mosquito, *Culex quinquefasciatus* (Diptera: Culicidae).

MB elicited a weak attractive effect against most insect species tested here, except for *T. castaneum.* In contrast, AP showed weak repellency against *R. dominica*, *T. castaneum*, and *T. granarium* but exhibited an attractive effect toward *S. oryzae*. This agrees with previous findings showing that both compounds exhibited repellent and attractive effects against pests. For example, Xiao et al. [33] reported that MB showed weak repellency against *L. serricorne*, with RP% values of 29.4% and 41.9% at 10 and 4000 ppm, respectively. Liang et al. [49] found that AP derived from the essential oil of *Elsholtzia densa*, a mint plant in the family *Lamiaceae*, induced a significant repellent effect against *T. castaneum* and *L. serricorne* with RP% values > 90.0% at about 80.0 µg/cm^2^. AP was also found to repel the red palm weevil, *Rhynchophorus ferrugineus* (Olivier) (Coleoptera: Curculionidae) [59]. MB and AP exhibited repellent action against the bed bug, *Cimex lectularius* L. (Hemiptera: Cimicidae) [60]. Moreover, MB and AP acted as attractants for pests in previous studies, supporting our present results. For instance, Wang et al. [61,62] reported that AP exhibited a significant attractive effect against the Indian meal moth, *Plodia interpunctella* (Hubner, 1813) (Lepidoptera: Pyralidae). Gao et al. [63] noted that MB was more attractive to a greater number of the bark beetle, *Trypophloeus klimeschi* Eggers (Coleoptera: Curculionidae), than the control.

## 5. Conclusions

Methyl benzoate and AP exhibited significant toxicity as residual films and as wheat grain protectants against the four tested beetle species. Conversely, MB and AP exerted negligible to weak repellent and attractive effects toward these insects. Therefore, MB and AP could serve as promising candidates to protect stored grains against insect pests. Furthermore, future work could study both compounds being used on their own or in combination with other grain-protective tools, including inert dusts and conventional insecticides. However, treating cereal grains with either compound studied here may affect the flavor and quality of food products processed from them, which deserves further investigation. Additionally, further research is needed to develop effective formulations for both compounds and to evaluate factors that may influence their performance, including temperature, relative humidity, and grain moisture content, as well as to assess their residual persistence and potential impacts on grain quality and seed germination.

## Figures and Tables

**Table 1:** Probit parameters for adult beetles exposed to different concentrations of methyl benzoate (MB) and acetophenone (AP) for 24 h at 25.0 °C.

Compound	Species	LC_50_ (mg/cm^2^) (95% Confidence Limits)	LC_99_ (mg/cm^2^) (95% Confidence Limits)	Slope ± SE	Intercept ± SE	(χ^2^)	*p* Value
MB	*R. dominica*	0.265 (0.241–0.292)	0.923 (0.741–1.259)	4.29 ± 0.40	2.48 ± 0.26	1.44	0.697
	*S. oryzae*	0.233 (0.211–0.260)	1.027 (0.771–1.567)	3.61 ± 0.37	2.28 ± 0.27	4.08	0.395
	*T. castaneum*	0.257 (0.232–0.284)	1.165 (0.908–1.653)	3.54 ± 0.32	2.09 ± 0.21	4.79	0.309
	*T. granarium*	0.149 (0.134–0.166)	0.598 (0.480–0.812)	3.86 ± 0.36	3.19 ± 0.29	5.29	0.152
AP	*R. dominica*	0.328 (0.284–0.363)	0.544 (0.466–0.774)	10.57 ± 1.01	5.12 ± 0.47	6.14	0.105
	*S. oryzae*	0.156 (0.107–0.212)	0.919 (0.499–5.228)	3.02 ± 0.32	2.44 ± 0.26	6.53	0.089
	*T. castaneum*	0.233 (0.209–0.261)	0.998 (0.759–1.487)	3.68 ± 0.37	2.33 ± 0.26	3.59	0.310
	*T. granarium*	0.126 (0.114–0.138)	0.399 (0.330–0.522)	4.63 ± 0.44	4.17 ± 0.39	0.39	0.943

**Table 2:** ANOVA parameters for adult mortality of *R. dominica*, *S. oryzae*, *T. castaneum*, and *T. granarium* adults exposed to wheat grains treated with methyl benzoate (MB) and acetophenone (AP) for 3, 7, and 14 days at different application rates.

	Species	*R. dominica*			*S. oryzae*			*T. castaneum*			*T. granarium*		
	Source	df *	F	*p*	df	F	*p*	df	F	*p*	df	F	*p*
MB	Intercept	1.0	5186.84	<0.001	1.0	676.61	<0.001	1.0	34,907.60	<0.001	1.0	1712.16	<0.001
	Rate	3.0	223.46	<0.001	3.0	25.13	<0.001	3.0	81.54	<0.001	3.0	26.76	<0.001
	Time	1.0	1.0	0.347	1.0	2.13	0.181	-	-	-	1.0	2.54	0.15
	Rate × Time	3.0	1.0	0.441	3.0	1.40	0.310	-	-	-	3.0	2.54	0.13
AP	Intercept	1.0	927.68	<0.001	1.0	1095.76	<0.001	1.0	1655.37	<0.001	1.0	1038.02	<0.001
	Rate	3.0	247.67	<0.001	3.0	100.48	<0.001	3.0	50.43	<0.001	3.0	27.57	<0.001
	Time	1.0	3.84	0.086	1.0	22.73	<0.001	2.0	4.51	0.028	1.0	30.19	<0.001
	Rate × Time	3.0	3.84	0.057	3.0	18.62	<0.001	6.0	1.92	0.138	3.0	17.49	<0.001

* The Greenhouse–Geisser correction was applied, except for AP against *T. castaneum*, where the sphericity assumption was met (Mauchly’s test, *p* > 0.05).

**Table 3:** Mortality percentages (mean ± SE) of *R. dominica*, *S. oryzae*, *T. castaneum*, and *T. granarium* adults exposed to wheat grains treated with methyl benzoate for 3, 7, and 14 days at different application rates.

Insect (Application Rate, g/kg)	Mortality (Mean % ± SE) *		
	3 Days	7 Days	14 Days
*R. dominica*			
0.25	21.7 ± 6.6 b	23.3 ± 6.0 b	23.3 ± 6.0 b
0.5	100.0 ± 0.0 a	100.0 ± 0.0 a	100.0 ± 0.0 a
1.0	100.0 ± 0.0 a	100.0 ± 0.0 a	100.0 ± 0.0 a
2.0	100.0 ± 0.0 a	100.0 ± 0.0 a	100.0 ± 0.0 a
Significance	F (3, 8) = 194.7, *p* < 0.001	F (3, 8) = 234.2, *p* < 0.001	F (3, 8) = 234.2, *p* < 0.001
*S. oryzae*			
0.25	28.3 ± 7.6 b	30.0 ± 9.4 b	27.5 ± 9.8 b
0.5	86.8 ± 8.2 a	86.8 ± 8.2 a	86.3 ± 8.5 a
1.0	100.0 ± 0.0 a	100.0 ± 0.0 a	100.0 ± 0.0 a
2.0	100.0 ± 0.0 a	100.0 ± 0.0 a	100.0 ± 0.0 a
Significance	F (3, 8) = 27.5, *p* < 0.001	F (3, 8) = 23.9, *p* < 0.001	F (3, 8) = 23.9, *p* < 0.001
*T. castaneum*			
0.25	91.7 ± 1.7 b	91.7 ± 1.7 b	91.7 ± 1.7 b
0.5	100.0 ± 0.0 a	100.0 ± 0.0 a	100.0 ± 0.0 a
1.0	100.0 ± 0.0 a	100.0 ± 0.0 a	100.0 ± 0.0 a
2.0	100.0 ± 0.0 a	100.0 ± 0.0 a	100.0 ± 0.0 a
Significance	F (3, 8) = 81.5, *p* < 0.001	F (3, 8) = 81.5, *p* < 0.001	F (3, 8) = 81.5, *p* < 0.001
*T. granarium*			
0.25	45.7 ± 4.3 b	67.4 ± 9.9 b	NT
0.5	95.7 ± 4.3 a	95.7 ± 4.3 a	NT
1.0	100.0 ± 0.0 a	100.0 ± 0.0 a	NT
2.0	100.0 ± 0.0 a	100.0 ± 0.0 a	NT
Significance	F (3, 8) = 37.2, *p* < 0.001	F (3, 8) = 11.3, *p* < 0.001	

* Mean values followed by the same letter within a column within each insect species are not significantly different according to Bonferroni-adjusted pairwise comparisons (*p* > 0.05). NT: not tested.

**Table 4:** Mortality percentages (mean ± SE) of *R. dominica*, *S. oryzae*, *T. castaneum*, and *T. granarium* adults exposed to wheat grains treated with acetophenone for 3, 7, and 14 days at different application rates.

Insect (Application Rate, g/kg)	Mortality (Mean % ± SE) *		
	3 Days	7 Days	14 Days
*R. dominica*			
0.25	0.0 ± 0.0 c	0.0 ± 0.0 c	0.0 ± 0.0 c
0.5	5.0 ± 2.9 c	5.0 ± 2.9 c	5.0 ± 2.9 c
1.0	88.3 ± 1.7 b	91.7 ± 1.7 b	91.7 ± 1.7 b
2.0	100.0 ± 0.0 a	100.0 ± 0.0 a	100.0 ± 0.0 a
Significance	F (3, 8) = 244.2, *p* < 0.001	F (3, 8) = 242.4, *p* < 0.001	F (3, 8) = 242.4, *p* < 0.001
*S. oryzae*			
0.25	3.1 ± 3.1 c	5.0 ± 5.0 b	5.9 ± 5.9 b
0.5	67.9 ± 1.9 b	96.2 ± 1.9 a	96.0 ± 1.9 a
1.0	100.0 ± 0.0 a	100.0 ± 0.0 a	100.0 ± 0.0 a
2.0	100.0 ± 0.0 a	100.0 ± 0.0 a	100.0 ± 0.0 a
Significance	F (3, 8) = 171.8, *p* < 0.001	F (3, 8) = 80.6, *p* < 0.001	F (3, 8) = 69.2, *p* < 0.001
*T. castaneum*			
0.25	36.6 ± 7.2 c	36.6 ± 7.2 c	41.7 ± 4.4 c
0.5	75.0 ± 5.7 b	76.6 ± 6.0 b	78.3 ± 4.4 b
1.0	98.3 ± 1.7 a	98.3 ± 1.7 a	98.3 ± 1.7 a
2.0	100.0 ± 0.0 a	100.0 ± 0.0 a	100.0 ± 0.0 a
Significance	F (3, 8) = 45.8, *p* < 0.001	F (3, 8) = 44.6, *p* < 0.001	F (3, 8) = 61.7, *p* < 0.001
*T. granarium*			
0.25	19.6 ± 9.5 c	78.3 ± 2.2 bc	NT
0.5	56.5 ± 5.7 b	71.8 ± 5.7 c	NT
1.0	93.5 ± 3.8 a	93.5 ± 3.8 ab	NT
2.0	100.0 ± 0.0 a	100.0 ± 0.0 a	NT
Significance	F (3, 8) = 29.9, *p* < 0.001	F (3, 8) = 15.8, *p* < 0.001	

* Mean values followed by the same letter within a column within each insect species are not significantly different according to Bonferroni-adjusted pairwise comparisons (*p* > 0.05). NT: not tested.

**Table 5:** Emerged adults (mean ± SE) of *R. dominica*, *S. oryzae*, *T. castaneum*, and *T. granarium* and reduction % after 65 days from exposure to wheat treated with methyl benzoate and acetophenone at different application rates.

Insects (Application Rate, g/kg)	Methyl Benzoate		Acetophenone	
	Emerged Adults (Mean ± SE) *	Reduction %	Emerged Adults (Mean ± SE) *	Reduction %
*R. dominica*				
0.0	175.3 ± 16.5 a	-	175.3 ± 16.5 a	-
0.25	80.3 ± 10.6 b	54.2	86.3 ± 25.0 b	50.8
0.5	0.0 ± 0.0 c	100.0	42.3 ± 14.0 bc	75.9
1.0	0.0 ± 0.0 c	100.0	18.3 ± 15.8 bc	89.6
2.0	0.0 ± 0.0 c	100.0	0.0 ± 0.0 c	100.0
Significance	F (4, 10) = 77.9, *p* < 0.001		F (4, 10) = 18.0, *p* < 0.001	
*S. oryzae*				
0.0	160.7 ± 16.4 a	-	160.7 ± 16.4 a	-
0.25	92.3 ± 26.4 b	42.6	27.7 ± 9.7 b	82.8
0.5	4.7 ± 2.6 c	97.1	0.0 ± 0.0 b	100.0
1.0	0.0 ± 0.0 c	100.0	0.0 ± 0.0 b	100.0
2.0	0.0 ± 0.0 c	100.0	0.0 ± 0.0 b	100.0
Significance	F (4, 10) = 27.2, *p* < 0.001		F (4, 10) = 67.1, *p* < 0.001	
*T. castaneum*				
0.0	27.3 ± 5.2 a	-	27.3 ± 5.2 a	-
0.25	0.0 ± 0.0 b	100.0	0.0 ± 0.0 b	100.0
0.5	0.0 ± 0.0 b	100.0	0.0 ± 0.0 b	100.0
1.0	0.0 ± 0.0 b	100.0	0.0 ± 0.0 b	100.0
2.0	0.0 ± 0.0 b	100.0	0.0 ± 0.0 b	100.0
Significance	F (4, 10) = 27.9, *p* < 0.001		F (4, 10) = 27.9, *p* < 0.001	
*T. granarium*				
0.0	259.7 ± 29.6 a	-	259.7 ± 29.6 a	-
0.25	0.0 ± 0.0 b	100.0	1.3 ± 0.7 b	99.5
0.5	0.0 ± 0.0 b	100.0	0.0 ± 0.0 b	100.0
1.0	0.0 ± 0.0 b	100.0	0.0 ± 0.0 b	100.0
2.0	0.0 ± 0.0 b	100.0	0.0 ± 0.0 b	100.0
Significance	F (4, 10) = 77.2, *p* < 0.001		F (4, 10) = 76.9, *p* < 0.001	

* Mean values followed by the same letter within a column within each insect species are not significantly different. (*p* > 0.05) according to ANOVA followed by the Tukey HSD test.

**Table 6:** ANOVA parameters for repellency index of methyl benzoate (MB) and acetophenone (AP) against *R. dominica*, *S. oryzae*, *T. castaneum*, and *T. granarium* adults at different application rates and different exposure times.

	Species	*R. dominica*			*S. oryzae*			*T. castaneum*			*T. granarium*		
	Source	df	F	*p*	df	F	*p*	df	F	*p*	df	F	*p*
MB	Intercept	1.0 *	510.8	<0.001	1.0	2722.7	<0.001	1.0	71.94	<0.001	1.0	314.26	<0.001
	Rate	3.0	6.31	0.017	3.0	9.49	0.005	3.0	0.27	0.848	3.0	0.67	0.591
	Time	1.2	2.21	0.169	2.0	10.28	0.001	2.0	3.55	0.053	2.0	1.24	0.315
	Rate x Time	3.57	1.82	0.206	6.0	0.92	0.508	6.0	1.42	0.266	6.0	1.02	0.447
AP	Intercept	1.0	192.36	<0.001	1.0	901.14	<0.001	1.0	17.33	0.003	1.0	349.92	<0.001
	Rate	3.0	0.29	0.828	3.0	0.85	0.504	3.0	0.13	0.937	3.0	8.08	0.008
	Time	2.0	0.03	0.969	2.0	23.67	<0.001	2.0	3.06	0.075	2.0	0.12	0.888
	Rate x Time	6.0	0.37	0.885	6.0	5.21	0.004	6.0	3.00	0.037	6.0	0.35	0.901

* The Greenhouse–Geisser correction was applied.

**Table 7:** The repellency index (RI) (mean ± SE) of methyl benzoate against adults of *R. dominica*, *T. castaneum*, *S. oryzae*, and *T. granarium* at different application rates and after different exposure times.

Insect (Application Rate, µg/cm^2^)	RI (Mean ± SE) *			Overall RI (Mean ± SE)	Classification
	0.5 h	1.0 h	2.0 h		
*R. dominica*					
6.25	0.83 ± 0.13 a	0.63 ± 0.12 a	0.67 ± 0.18 b	0.71 ± 0.07 b	Repellent
12.5	1.13 ± 0.12 a	1.16 ± 0.20 a	1.47 ± 0.03 a	1.26 ± 0.09 a	Attractive
25.0	0.87 ± 0.18 a	1.23 ± 0.32 a	1.07 ± 0.07 ab	1.06 ± 0.12 ab	Attractive
50.0	0.70 ± 0.10 a	1.46 ± 0.24 a	1.10 ± 0.10 ab	1.09 ± 0.41 ab	Attractive
Significance	F (3, 8) = 1.80, *p* = 0.225	F (3, 8) = 2.31, *p* = 0.153	F (3, 8) = 9.17, *p* = 0.006	F (3, 8) = 6.31, *p* = 0.017	
*S. oryzae*					
6.25	0.87 ± 0.08 a	1.53 ± 0.03 a	1.15 ± 0.07 b	1.18 ± 0.11 b	Attractive
12.5	1.17 ± 0.09 a	1.43 ± 0.19 a	1.10 ± 0.15 b	1.23 ± 0.09 b	Attractive
25.0	1.27 ± 0.03 a	1.60 ± 0.11 a	1.23 ± 0.09 ab	1.37 ± 0.07 ab	Attractive
50.0	1.27 ± 0.24 a	1.70 ± 0.10 a	1.63 ± 0.09 a	1.53 ± 0.10 a	Attractive
Significance	F (3, 8) = 1.92, *p* = 0.204	F (3, 8) = 0.86, *p* = 0.502	F (3, 8) = 5.33, *p* = 0.026	F (3, 8) = 9.94, *p* = 0.005	
*T. castaneum*					
6.25	0.80 ± 0.0 a	0.87 ± 0.38 a	1.00 ± 0.20 a	0.89 ± 0.13 a	Repellent
12.5	1.10 ± 0.25 a	0.53 ± 0.20 a	0.50 ± 0.11 a	0.71 ± 0.14 a	Repellent
25.0	1.10 ± 0.38 a	0.90 ± 0.18 a	0.60 ± 0.23 a	0.87 ± 0.16 a	Repellent
50.0	0.87 ± 0.32 a	0.83 ± 0.12 a	0.43 ± 0.12 a	0.71 ± 0.12 a	Repellent
Significance	F (3, 8) = 0.31, *p* = 0.817	F (3, 8) = 0.49, *p* = 0.70	F (3, 8) = 2.07, *p* = 0.182	F (3, 8) = 0.27, *p* = 0.848	
*T. granarium*					
6.25	1.27 ± 0.21 a	1.25 ± 0.19 a	1.13 ± 0.28 a	1.22 ± 0.12 a	Attractive
12.5	1.38 ± 0.17 a	1.33 ± 0.09 a	1.30 ± 0.21 a	1.34 ± 0.08 a	Attractive
25.0	1.17 ± 0.09 a	1.43 ± 0.03 a	1.43 ± 0.09 a	1.34 ± 0.06 a	Attractive
50.0	0.97 ± 0.21 a	1.23 ± 0.09 a	1.10 ± 0.11 a	1.10 ± 0.08 a	Attractive
Significance	F (3, 8) = 0.94, *p* = 0.464	F (3, 8) = 0.64, *p* = 0.610	F (3, 8) = 0.66, *p* = 0.600	F (3, 8) = 0.67, *p* = 0.591	

* Mean values followed by the same letter within a column within each insect species are not significantly different according to Bonferroni-adjusted pairwise comparisons (*p* > 0.05).

**Table 8:** The repellency index (RI) (mean ± SE) of acetophenone against adults of *R. dominica*, *T. castaneum*, *S. oryzae*, and *T. granarium* at different application rates and after different exposure times.

Insect (Application Rate, µg/cm^2^)	RI (Mean ± SE) *			Overall RI (Mean ± SE) *	Classification
	0.5 h	1.0 h	2.0 h		
*R. dominica*					
6.25	0.70 ± 0.21 a	0.53 ± 0.17 a	0.50 ± 0.11 a	0.57 ± 0.09 a	Repellent
12.5	0.60 ± 0.15 a	0.67 ± 0.03 a	0.63 ± 0.07 a	0.63 ± 0.05 a	Repellent
25.0	0.73 ± 0.15 a	0.67 ± 0.03 a	0.70 ± 0.06 a	0.70 ± 0.05 a	Repellent
50.0	0.53 ± 0.15 a	0.73 ± 0.29 a	0.67 ± 0.15 a	0.64 ± 0.11 a	Repellent
Significance	F (3, 8) = 0.31, *p* = 0.818	F (3, 8) = 0.24, *p* = 0.867	F (3, 8) = 0.73, *p* = 0.563	F (3, 8) = 0.29, *p* = 0.828	
*S. oryzae*					
6.25	1.60 ± 0.11 a	1.33 ± 0.12 a	0.93 ± 0.07 a	1.29 ± 0.11 a	Attractive
12.5	1.43 ± 0.07 a	1.83 ± 0.03 a	1.20 ± 0.15 a	1.49 ± 0.11 a	Attractive
25.0	1.50 ± 0.10 a	1.47 ± 0.15 a	1.20 ± 0.11 a	1.39 ± 0.08 a	Attractive
50.0	1.60 ± 0.00 a	1.37 ± 0.15 a	1.37 ± 0.18 a	1.44 ± 0.08 a	Attractive
Significance	F (3, 8) = 0.96, *p* = 0.457	F (3, 8) = 3.64, *p* = 0.064	F (3, 8) = 1.78, *p* = 0.229	F (3, 8) = 0.85, *p* = 0.504	
*T. castaneum*					
6.25	0.77 ± 0.33 a	0.50 ± 0.3 a	0.40 ± 0.1 a	0.56 ± 0.14 a	Repellent
12.5	0.20 ± 0.11 a	0.97 ± 0.55 a	1.03 ± 0.43 a	0.73 ± 0.24 a	Repellent
25.0	0.47 ± 0.22 a	0.47 ± 0.41 a	0.53 ± 0.53 a	0.49 ± 0.21 a	Repellent
50.0	0.33 ± 0.12 a	0.57 ± 0.19 a	1.03 ± 0.03 a	0.64 ± 0.12 a	Repellent
Significance	F (3, 8) = 1.28, *p* = 0.345	F (3, 8) = 0.36, *p* = 0.785	F (3, 8) = 0.91, *p* = 0.478	F (3, 8) = 0.13, *p* = 0.937	
*T. granarium*					
6.25	1.07 ± 0.18 a	1.27 ± 0.22 a	1.20 ± 0.11 a	1.18 ± 0.09 a	Attractive
12.5	1.00 ± 0.06 a	1.00 ± 0.0 ab	1.03 ± 0.03 a	1.01 ± 0.06 a	Attractive
25.0	0.90 ± 0.06 a	0.87 ± 0.09 ab	0.87 ± 0.27 a	0.88 ± 0.08 ab	Repellent
50.0	0.60 ± 0.15 a	0.57 ± 0.07 c	0.43 ± 0.18 a	0.53 ± 0.07 b	Repellent
Significance	F (3, 8) = 2.78, *p* = 0.110	F (3, 8) = 5.65, *p* = 0.022	F (3, 8) = 3.61, *p* = 0.065	F (3, 8) = 8.08, *p* = 0.008	

* Mean values followed by the same letter within a column within each insect species are not significantly different according to Bonferroni-adjusted pairwise comparisons (*p* > 0.05).

## Data Availability

The data in the present study are available on request from the corresponding authors.
